# Why is “early childhood intensive care” an Italian association of neonatology study group?

**DOI:** 10.1186/s13052-019-0626-x

**Published:** 2019-03-06

**Authors:** Nicola Pozzi, Gabriella D’Angelo, Eloisa Gitto, Gruppo di Studio Terapia Intensiva della Prima Infanzia della SIN

**Affiliations:** 1 Neonatal and Pediatric Intensive Care Unit, Maternal and Child Department, San Pio Hospital, Benevento, Italy; 2 Neonatal and Pediatric Intensive Care Unit, Department of Human Pathology in Adult and Developmental Age “Gaetano Barresi”, University of Messina, Via Consolare Valeria, 1, 98125 Messina, Italy; 3 Department of Clinical and Experimental Medicine, University of Messina, Via Consolare Valeria, 1, 98125 Messina, Italy

**Keywords:** Critically ill children, Newborn, Neonatal intensive care unit, Pediatric intensive therapy

## Abstract

To date, a large number of children are hospitalized inappropriately in adult intensive care units, where the minimum standards of care are not applied to young patients. It is well-known that the child is not a small adult. Recently it has been demonstrated that critically ill children hospitalized in pediatric intensive care receive higher quality of care, and have better outcomes, besides a lower mortality rate, compared to those admitted to adult intensive care units.

We believe that the management of the critically ill child is an area of expertise of the neonatologist, who however must acquire specific skills and abilities of pediatric intensive medicine. The new idea of care is to offer in general hospitals ‘broader’ Neonatal Intensive Care Units, extended to infants and children in early childhood, based on territorial macro-areas and/or population of competence.

The World Health Organization (WHO) reported that in 2017 about 4.1 million children under the age of 5 died, 75% of whom were infants. In Italy, again in 2017, the mortality rate under 1 year of life was 2.9 per 1000 live births compared to a neonatal mortality rate of 2.0 per 1000 live births [1]. What can be done to improve this situation?

In recent years, several studies have shown that critically ill children hospitalized in pediatric intensive care units (PICUs) receive higher quality of care, and have better outcomes, besides a lower mortality rate, compared to those admitted to adult intensive care units, both in Italy [2] and in developing countries [3]. There are 23 PICUs, which were surveyed in 2014, on the national territory, but half are distributed in the North of Italy, 8 are in the Center and just 4 in the South (Fig. 1). The consequence is that a large number of children are hospitalized inappropriately in adult intensive care units, where the minimum standards of care are not applied to young patients.

**Fig. 1:**
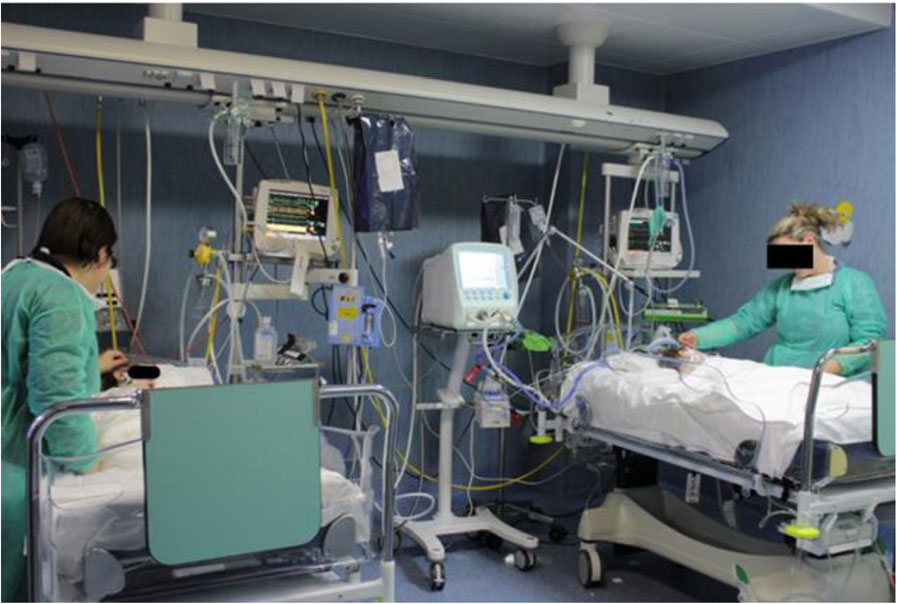
Critilcally ill children hospitalized and managed in a Neonatal and Pediatric Intensive Care Unit in San Pio Hospital (Benevento)

It is well-known that the child is not a small adult. However, in recent years in Italy the number of beds dedicated to pediatric intensive cares have not increased, in contrast with what has happened in other European countries and North America, with significant discrepancies between the north and south of our country (ratio of beds/children: USA 1:18,000, UK 1:25,000, IT 1:75,000, (IT North 1:50,000, IT South 1:150,000)) [4]. Moreover, from the data of the Italian Pediatric Intensive Care Units Network (TIPNet), it emerges that children hospitalized in PICUs have a mean age of 45.4 ± 54.5 months (mean ± SD) and about 70% are in the pre-school age group (0–5 years), very similar to the period of early childhood. About 30% are children under the age of one. In addition, about 55% of the children were admitted to the PICUs only for medical reasons and among them about 50% for acute respiratory problems [4].

However, improvement over the years in the quality of care in our Neonatal Intensive Care Units (NICUs) has led to an increase in the survival of medically complex compromised children, including children with extreme prematurity, with congenital malformations or suffering from chronic, often respiratory diseases, with neuromuscular diseases or with severe neurological disabilities from perinatal asphyxia [5]. Often the situation of the lack of intensive pediatric beds is exacerbated by the high rate of employment by long-term chronic children, who represent about 40% of the total hospitalizations in PICUs in the age group < 5 years [4], with related low turn-over of acute places.

To solve the chronic shortage of pediatric intensive beds and taking into account that a good share of critically ill children fall into the under-1 year age group and are mainly affected by medical diseases (respiratory or septic nature, similar to those usually managed by neonatologists), it is reasonable to think that some of the NICUs, present widely in the national territory even in areas where there is a low prevalence of PICUs, can be organized to manage acute or chronic-exacerbated infants and babies.

In this direction, it is also interesting to underline the experiences of others European countries, including Denmark, where the management of critically ill children under the age of 1 year has been centralized in a NICU of the east of the country, covering a territory about 2.5 million to rationalize resources and delivering a care comparable to international standards [6].

Therefore, the *new idea of care* is to offer in general hospitals ‘broader’ Neonatal Intensive Care Units, extended to infants and children in early childhood, based on territorial macro-areas and/or population of competence. Thus, pediatric hospitals will bring together children suffering from complex pathologies requiring high level specializations (neonatal/pediatric surgery, cardiac surgery, traumatology, neurosurgery, large burns center, etc.) and into a regional nodal network which is sub-divided by levels of assistance. This is the concept of *centralization of pediatric intensive cares* that would also allow to acquire and maintain various technical skills for the correct management of pediatric critically ill patients.

Many aspects of modern neonatal care resemble those of pediatric intensive medicine but, on the other hand, there are several clinical and management features of the critically ill child that are unique. This implies that the neonatologist has to acquire a sufficient level of knowledge and clinical expertise in both the neonatal and pediatric fields to manage critically ill children in a safe way, following international standards [7].

According to a recent questionnaire on “Enlarged NICU” sent to all Italian Association of Neonatology (SIN) members by our study group, it emerged that in Italy, in 66% of the 92 NICUs that responded to the survey, children older than 1 month of life are currently hospitalized and managed in these units. Moreover, in 78% of cases, respiratory disease was the most frequent cause of hospitalization. Some centers have already reached an excellent level of organization and treatment of pediatric intensive diseases.

Therefore, we believe that the management of the critically ill child is certainly an area of expertise of the neonatologist, who however must acquire specific skills and abilities of pediatric intensive medicine and will need specific programs of multidisciplinary training in PICUs.

In this context, SIN’s “Early Childhood Intensive Care” (TIPI) study group wants to promote among all members the knowledge of critically ill infants and the subsequent training of all interested neonatologists in management of critically ill children in their NICUs.

## Abbreviations

NICUs: Neonatal Intensive Care Units
PICU: Pediatric Intensive Care Units
SIN: Italian Association of Neonatology
TIPI: Early Childhood Intensive Care
TIPNet: Italian Pediatric Intensive Care Units Network
WHO: World Health Organization
